# Tetranortriterpenes and Limonoids from the Roots of *Aphanamixis polystachya*

**DOI:** 10.3390/molecules21091167

**Published:** 2016-09-02

**Authors:** Ching-Jie Lin, I-Wen Lo, Yu-Chi Lin, Shun-Ying Chen, Ching-Te Chien, Yao-Haur Kuo, Tsong-Long Hwang, Shorong-Shii Liou, Ya-Ching Shen

**Affiliations:** 1 School of Pharmacy, College of Medicine, National Taiwan University, No. 33, Linsen S. Rd., Zhongzheng Dist., Taipei 10050, Taiwan; r00423014@ntu.edu.tw (C.-J.L.); d96423006@ntu.edu.tw (I.-W.L.); 2 Department of Life Sciences, National Cheng Kung University, No. 1, University Road, Tainan 701, Taiwan; z10108042@email.ncku.edu.tw; 3 Taiwan Forestry Research Institute, Council of Agriculture, Executive Yuan, No. 53, Nanhai Rd., Zhongzheng Dist., Taipei 10066, Taiwan; sychen@tfri.gov.tw (S.-Y.C.); chien@tfri.gov.tw (C.-T.C.); 4 National Research Institute of Chinese Medicine, Ministry of Health and Welfare, No. 155-1, Sec. 2, Linong St., Beitou District, Taipei 11221, Taiwan; kuoyh@nricm.edu.tw; 5 Graduate Institute of Natural Products, School of Traditional Medicine, College of Medicine, and Chinese Herbal Medicine Research Team, Healthy Aging Research Center, Chang Gung University, Taoyuan 33302, Taiwan; htl@mail.cgu.edu.tw; 6 Department of Cosmetic Science and Research Center for Industry of Human Ecology, Chang Gung University of Science and Technology, Taoyuan 33302, Taiwan; 7 Department of Pharmacy, Tajen University, No. 20, Weixin Rd., Yanpu Township, Pingtung 90741, Taiwan; ssliou@tajen.edu.tw

**Keywords:** *Aphanamixis polystachya*, apotirucallane-type triterpenoid, limonoid, cytotoxicity, anti-inflammatory activity

## Abstract

Phytochemical investigation of the acetone extract from the roots of *Aphanamixis polystachya* resulted in isolation of four new tetranortriterpenes (**1**–**4**) in addition to one protolimonoid (methyl-1ξ,7*R*-diacetoxy-23*R*,25-dihydroxy-20*S*,24*R*-21,24-epoxy-3,4-seco-apotirucall-4(28),14(15)-diene-3-oate (**5**)), five known limonoids (rohituka 3 (**6**), rohituka 7 (**7**), nymania 1 (**8**), rubrin G (**9**), prieurianin (**10**)) and a steroid (2,3-dihydroxy-5-pregnan-16-one (**11**)). Their structures were determined by spectroscopic analyses, including 2D-NMR (COSY, HMQC, HMBC, and NOESY) and high-resolution electrospray ionization mass spectrometry (HRESIMS). Cytotoxic and anti-inflammatory activities of these compounds were evaluated. Compounds **4** and **5** showed significant inhibition against superoxide generation and elastase release by human neutrophils in response to (formyl-l-methionyl-l-leucyl-l-phenylalanine/cytochalasin B) (FMLP/CB).

## 1. Introduction

*Aphanamixis polystachya* (Wall.) R.N. Parker (Meliaceae) is a tropical neem tree native to Asia, especially China and India. According to previous studies, the meliaceaeous plants are famous for the limonoid-producing source and the pest control function in agriculture [1,2]. Phytochemical investigations of this plant have resulted in isolation of many limonoids with diverse skeletons [3,4,5,6,7]. Other structures such as guaiane sesquiterpenes from the seeds [8], lignans from the stem barks [9], chromone, and flavonoid glycosides from the roots [10] were also reported. In biological studies, it has been used as traditional Bangladesh medicine for the treatment of cancers, diabetes, and liver diseases [11] in addition to insecticide applications. Besides, the leaf extract showed CNS (central nervous system) depressant and analgesic activity in vivo [12], as well as antimicrobial, antioxidant, cytotoxic, and thrombolytic activities in vitro [13]. Currently, some limonoids from *A. polystachya* attract much attention due to their interesting anticancer [14], antifungal [15], and antifeedant activities [16,17].

Continuing our phytochemical investigation on the new anti-cancer and anti-inflammatory agents from terrestrial source, the crude extract from the roots of *A. polystachya* showed significant inhibition on superoxide anion generation and elastase release by human neutrophils in response to FMLP/CB at 10 μg/mL. Fractionation of the active components from the roots of this species was thus initiated. Herein, we report the isolation and structural elucidation of four new and six known triterpenoids, together with one known steroid from the roots of *A. polystachya*. The evaluation against human tumor cell lines and inhibitory activities on superoxide anion generation and elastase release are also discussed.

## 2. Materials and Methods

### 2.1. General Experimental Procedures

Optical rotations were recorded on a JASCO DIP-1000 polarimeter (JASCO, Tokyo, Japan). IR spectra were taken on a HORIBA FT-720 spectrophotometer (HORIBA, Kyoto, Japan). The ^1^H- and ^13^C-NMR spectra as well as 2D NMR spectra (COSY, HMQC, HMBC, and NOESY) were recorded in CDCl_3_ on a Bruker AVX NMR spectrometer (Bruker, Karlsruhe, Germany) operating at 400 MHz for ^1^H and 100 MHz for ^13^C using the CDCl_3_ solvent peak as internal standard (δ_H_ 7.265, δ_C_ 77.0 ppm). Low-resolution ESIMS were recorded on a VG Quattro 5022 mass spectrometer (VG Instruments, Altrincham, UK). HRESIMS were measured on a JEOL HX 110 mass spectrometer (JEOL, Peabody, MA, USA). LiChrospher^®^ Si 60 (5 μm, 250-10, Merck, Darmstadt, Germany) and LiChrospher^®^ 100 RP-18e (5 μm, 250-10, Merck) were used for NP-HPLC and RP-HPLC (Hitachi, L-6250, Kyoto, Japan; flow rate 2 mL/min, UV detection at 254 nm), respectively. HepG 2, A549 and MCF-7 cancer cells were purchased from Bioresource Collection and Research Center, Food Industry Research and Development Institute (Hsinchu, Taiwan), and Hep-2 was purchased from ATCC (American Type Culture Collectio, Manassas, VA, USA).

### 2.2. Plant Material

The roots of *Aphanamixis polystachya* (Wall.) R.N. Parker were collected in Ping-Tong County, Taiwan, in April 2012. The plant material was identified by one of the authors (C.T.C.). A voucher specimen (code No. TP 98-1) has been deposited at the School of Pharmacy, National Taiwan University, Taipei, Taiwan.

### 2.3. Extraction and Isolation

The air-dried roots (6.6 kg) of *A. polystachya* were ground and extracted thrice with acetone at room temperature and concentrated under reduced pressure to obtain a crude extract (250 g). The extract was partitioned between EtOAc:H_2_O (1:1) to give an EtOAc-soluble layer. After evaporating the organic solvent, the EtOAc residue (90 g) was partitioned between *n*-hexane:MeOH:H_2_O (4:3:1) to afford a MeOH/H_2_O extract. The MeOH/H_2_O extract (60 g) was subjected to pass over a Sephadex LH-20 column eluted with MeOH to afford fractions A–F. Fraction C (38 g) was separated on a Si gel (63–200 μm, 90 × 120 mm) column (*n*-hexane:EtOAc:MeOH, 100:0:0 to 0:0:100, 1 L for each gradient solvent) to afford fraction C-14 (7.1 g), which was further chromatographed on Si gel (<63 μm, 65 × 600 mm) column (*n*-hexane:EtOAc, 1:1, 5 L) to furnish three fractions, C-14-C, C-14-D, and C-14-E. Fraction C-14-C (1.7 g) was separated with a Si gel column and eluted with *n*-hexane:EtOAc:CH_2_Cl_2_ (1:1:1, 2.5 L) to give three subfractions, C-14-C-4, C-14-C-5 and C-14-C-6. Subfraction C-14-C-4 (385 mg) was subjected to RP-HPLC (5 μm, 10 mm × 250 mm) (MeOH:H_2_O, 7:1) to give **1** (7.6 mg, Rt = 20.3 min) and **2** (11.5 mg, Rt = 16.4 min). Subfraction C-14-C-6 (802 mg) was washed with MeOH and filtrated to yield **10** (443 mg). Fraction C-14-D (1.8 g) was separated by a reverse-phase column (20–50 μm, 35 × 35 mm) and eluted with MeOH/H_2_O (7:3, 2 L) to afford ten subfractions, C-14-D-1 to C-14-D-10. Subfraction C-14-D-6 (174 mg) was separated with an RP-HPLC (CH_3_CN/H_2_O, 55:45) to yield methyl-1ξ,7*R*-diacetoxy-23*R*,25-dihydroxy-20*S*,24*R*-21,24-epoxy-3,4-*seco*-apotirucall-4(28),14(15)-diene-3-oate (**5**, 15.6 mg, Rt = 35.4 min). Separation of fraction C-14-E (1.1 g) by a Si gel (<63 μm, 35 cm × 30 mm) column (*n*-hexane–acetone, 2:1, 2 L) obtained three subfractions, C-14-E-3, C-14-E-4 and C-14-E-6. Subfraction C-14-E-3 (236 mg) was applied on an RP-HPLC (CH_3_CN–H_2_O, 3:2) to yield **3** (18.1 mg, Rt = 39.1 min) and 2β,3β-dihydroxy-5α-pragnan-16-one (**11**, 17.8 mg, Rt = 22.2 min). Subfraction C-14-E-4 (187 mg) was purified by reverse-phase HPLC (MeOH–H_2_O, 7:1) to obtain **4** (26 mg, Rt = 11.1 min). Subfraction C-14-E-6 (358 mg) was separated by RP-HPLC (MeOH:H_2_O, 3:2) to furnish rohituka 3 (**6**, 30.7 mg, Rt = 37.7 min), rohituka 7 (**7**, 20.8 mg, Rt = 33.6 min), nymania 1 (**8**, 19.8 mg, Rt = 55.8 min), and rubrin G (**9**, 11.5 mg, Rt = 76.7 min).

## 3. Spectral Data

*Aphataiwanin A (***1***)*. Colorless amorphous solid; ${\left[\alpha \right]}_{D}^{25}$ −60 (*c* 0.1, MeOH); IR (CH_2_Cl_2_) *v*_max_ 3479, 3078, 1740, 1645, 977, 900 cm^−^^1^; ^1^H-NMR (CDCl_3_) and ^13^C-NMR (CDCl_3_) spectroscopic data, see Table 1 and Table 2, respectively; HRESIMS *m*/*z* 653.3684 [M + Na]^+^ (calcd for C_36_H_54_O_9_Na, 653.3660).

*Aphataiwanin B (***2***)*. Colorless amorphous solid; ${\left[\alpha \right]}_{D}^{25}$ −90 (*c* 0.1, MeOH); IR (CH_2_Cl_2_) *v*_max_ 3484, 3071, 1740, 1643, 983, 902 cm^−^^1^; ^1^H-NMR (CDCl_3_) and ^13^C-NMR (CDCl_3_) spectroscopic data, see Table 1 and Table 2, respectively; HRESIMS *m*/*z* 653.3681 [M + Na]^+^ (calcd for C_36_H_54_O_9_Na, 653.3660).

*Aphataiwanin C (***3***)*. Colorless amorphous solid; ${\left[\alpha \right]}_{D}^{25}$ −60 (*c* 0.1, MeOH); IR (CH_2_Cl_2_) *v*_max_ 3510, 2930, 1740, 1650, 972, 900 cm^−^^1^; ^1^H-NMR (CDCl_3_) and ^13^C-NMR (CDCl_3_) spectroscopic data, see Table 1 and Table 2, respectively; HRESIMS *m*/*z* 671.3785 [M + Na]^+^ (calcd for C_36_H_56_O_10_Na, 671.3765).

*Aphataiwanin D (***4***)*. Colorless amorphous solid; ${\left[\alpha \right]}_{D}^{25}$ −170 (*c* 0.1, MeOH); IR (CH_2_Cl_2_) *v*_max_ 3491, 2932, 1741, 1642, 900 cm^−^^1^; ^1^H-NMR (CDCl_3_) and ^13^C-NMR (CDCl_3_) spectroscopic data, see Table 1 and Table 2, respectively; HRESIMS *m*/*z* 671.3763 [M + Na]^+^ (calcd for C_36_H_56_O_10_Na, 671.3765).

## 4. Biological Activities

### 4.1. Cytotoxicity Assay

Cytotoxicity was tested against human Hep-G2 (hepatocellular carcinoma), HEp-2 (laryngeal carcinoma), A549 (lung carcinoma) and MCF-7 (breast adenocarcinoma) tumor cell lines. The assay procedure using MTT (3-(4,5-dimethylthiazole-2-yl)-2,5-diphenyltetrazolium bromide) was carried out as previously described [18,19]. The cells were cultured in RPMI-1640 medium. After seeding of cells in a 96-well microplate for 4 h, 20 μL of sample was placed in each well and incubated at 37 °C for 3 days, and then 20 μL MTT was added for 5 h. After removing the medium and putting DMSO (200 μL/well) into the microplate with shaking for 10 min, the formazan crystals were redissolved and their absorbance was measured on a microtiter plate reader (MR 7000, Dynatech, Missouri City, TX, USA) at a wavelength of 550 nm. Mitomycin C was used as a positive control.

### 4.2. Anti-Inflammatory Assays, Inhibitory Effect on Superoxide Anion Generation and Elastase Release by Human Neutrophils

Neutrophils were obtained by means of dextran sedimentation and Ficoll centrifugation. Superoxide generation and elastase release were carried out according to a procedure described previously [20]. Superoxide anion production was assayed by monitoring the superoxide dismutase-inhibitable reduction of ferricytochrome *c.* Elastase release experiments were performed using MeO-Suc-Ala-Ala-Pro-Valp-nitroanilide as the elastase substrate. Genistein was used as a standard compound, which showed inhibition of 65.0 ± 5.7 and 51.6 ± 5.9 at 10 μg/mL, respectively, on superoxide anion generation and elastase release.

## 5. Results and Discussion

**Figure 1:**
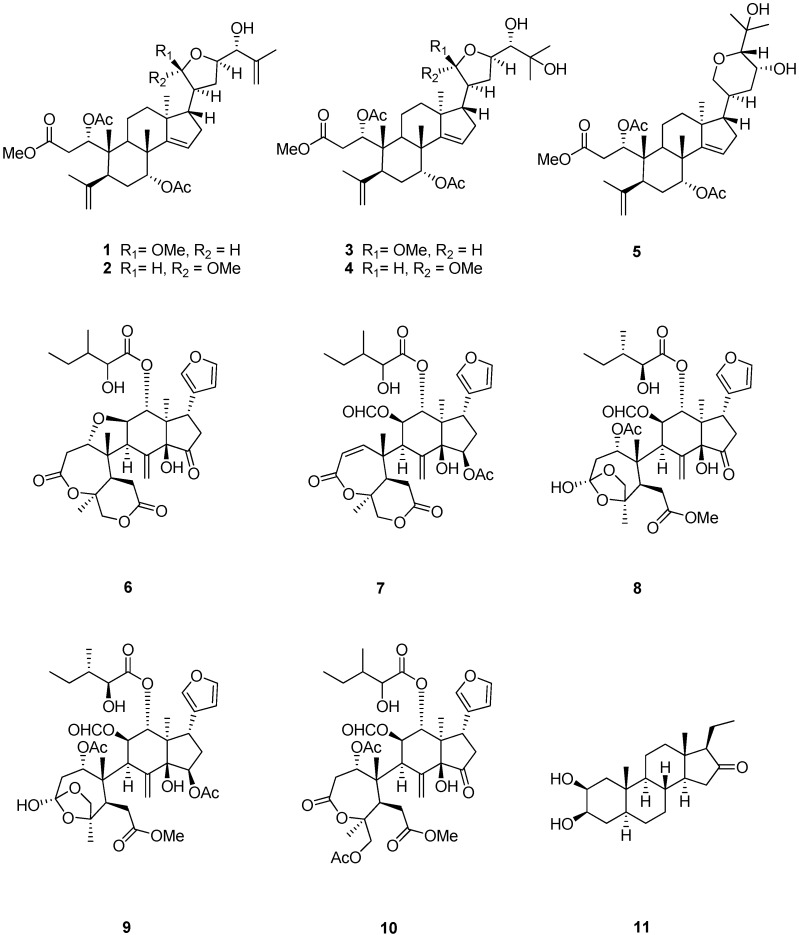
Chemical structures of compounds **1**–**11**.

Sephadex LH-20 and extensive column chromatography furnished four new apotirucallane-type triterpenoids **1**–**4** (Figure 1), together with a protolimonoid, methyl-1ξ,7*R*-diacetoxy-23*R*,25-dihydroxy-20*S*,24*R*-21,24-epoxy-3,4-*seco*-apotirucall-4(28),14(15)-diene-3-oate (**5**) [21]; five limonoids, rohituka 3 (**6**) [22], rohituka 7 (**7**) [22], nymania 1 (**8**) [23], rubrin G (**9**) [24], prieurianin (**10**) [25,26]; and one steroid, 2β,3β-dihydroxy-5α-pragnan-16-one (**11**) [27,28]. The structures of the new compounds were established by interpretation of their spectroscopic data, especially 2D NMR. The relative configurations of compounds **1**–**4** were determined by NOESY and comparison of NMR data with those published in reference papers.

Compound **1**, ${\left[\alpha \right]}_{D}^{25}$ −60 (MeOH), had the molecular formula of C_36_H_54_O_9_ and 10 degrees of unsaturation, as deduced from the HRESIMS (*m/z* 653.3684 [M + Na]^+^) and ^13^C-NMR/DEPT spectra. The IR absorption bands revealed the presence of hydroxy (3479 cm^−1^), ester (1740 cm^−1^), and double bond (1645 cm^−1^) functionalities in **1**. The ^1^H-NMR data of **1** (Table 1) exhibited seven methyl singlets (δ_H_ 0.96, 1.00, 1.14, 1.78, 1.79, 1.96 and 2.04), two methoxy singlets (δ_H_ 3.39, 3.66), exomethylene (δ_H_ 4.85, 4.93, 5.02 and 5.05), an olefinic methine doublet (δ_H_ 5.27, *J* = 3.0 Hz), and five oxygen-bearing methine signals (δ_H_ 3.83, 4.23, 4.78, 5.16 and 5.49). The ^13^C-NMR (Table 2) and DEPT spectra of **1** showed 36 carbon signals, consisting of three ester carbonyls (δ_C_ 170.2, 170.5, and 172.0), two olefinic methylenes (δ_C_ 112.8, 116.6), an olefinic methine (δ_C_ 119.1), three olefinic quaternary carbons (δ_C_ 144.9, 145.1, and 159.2), five oxymethines (δ_C_ 74.7, 77.2, 78.1, 80.7 and 104.6), four aliphatic methines (δ_C_ 34.5, 44.3, 45.8, and 52.9), six aliphatic methylenes (δ_C_ 18.5, 29.3, 31.0, 33.5, 35.2, and 35.5), two methoxyls (δ_C_ 52.3, 54.9), and seven methyls (δ_C_ 15.2, 18.7, 20.8, 21.2, 21.5, 23.0, and 26.9). This accounted for 6 of the 10 degrees of unsaturation, indicating that **1** is a tetracyclic triterpenoid with two acetyl and two methoxyl moieties.

**Figure 2:**
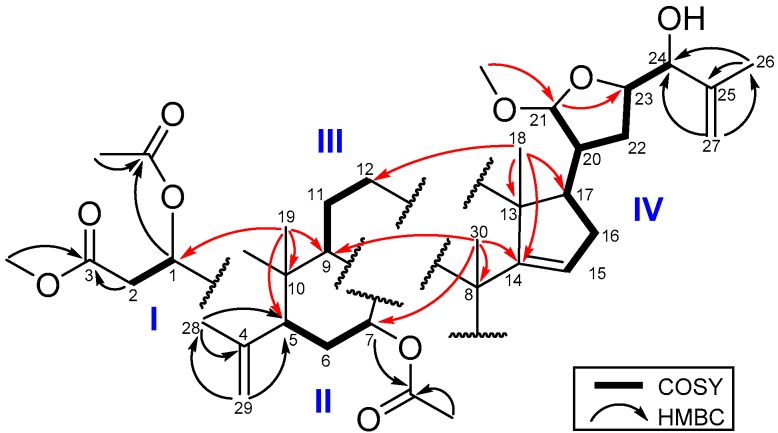
COSY and HMBC of **1**.

The COSY spectrum (Figure 2) of **1** exhibited four proton spin systems of H-1 (δ_H_ 5.49)/H_2_-2 (δ_H_ 2.47, 2.81) and H-5 (δ_H_ 2.44)/H_2_-6 (δ_H_ 1.63, 2.14)/H-7 (δ_H_ 5.16); H-9 (δ_H_ 2.18)/H_2_-11 (δ_H_ 1.67, and 1.89)/H_2_-12 (δ_H_ 1.54, and 1.65); and a proton sequence between H-15 (δ_H_ 5.27)/H_2_-16 (δ_H_ 1.93, 2.10)/H-17 (δ_H_ 1.92)/H-20 (δ_H_ 2.21)/H-21 (δ_H_ 4.78) and between H-20/H_2_-22 (δ_H_ 1.70, 1.87)/H-23 (δ_H_ 4.23) /H-24 (δ_H_ 3.83). In the HMBC spectrum (Figure 2), the H_3_-19 signal showed correlations to C-1 (δ_C_ 77.2), C-5 (δ_C_ 44.3), C-9 (δ_C_ 34.5) and C-10 (δ_C_ 44.3), and the H_2_-29 had correlations with C-4 (δ_C_ 145.1), C-5 and C-28 (δ_C_ 23.0) suggesting the ring B. In addition, the HMBC correlations of H_3_-18 (δ_H_ 1.00) with C-13 (δ_C_ 46.3), C-12 (δ_C_ 33.5), C-14 (δ_C_ 159.2), C-17 (δ_C_ 52.9), and of H_3_-30 (δ_H_ 1.14) with C-7 (δ_C_ 74.7), C-8 (δ_C_ 42.5), C-9 (δ_C_ 34.5) and C-14 (δ_C_ 159.2) established rings C and D. Moreover, the HMBC correlations of both H_3_-26 (δ_H_ 1.78) and H_2_-27 (δ_H_ 4.93, 5.05) with C-24 (δ_C_ 78.1) and C-25 (δ_C_ 144.9) indicated a methyl vinyl group to be attached at the oxygenated C-24. The HMBC correlations of the methoxy protons (δ_C_ 3.39) with C-21 (δ_C_ 104.6) and of H-21 (δ_H_ 4.78) with C-23 (δ_C_ 80.7) revealed the tetrahydrofuran ring with a methoxy group at C-21. The remaining methoxy (δ_H_ 3.36) group was found to be connected with C-3 (δ_C_ 172.0), and the H-1 (δ_H_ 5.49) and H-7 (δ_H_ 5.16) were connected with the acetyl carbonyl carbons (δ_C_ 170.5, 170.2) indicated that the acetyl groups were attached at C-1 and C-7, respectively. This 2D NMR spectroscopic analysis was used to identify **1** as an A-seco apo-tirucallane triterpenoid [15].

**Figure 3:**
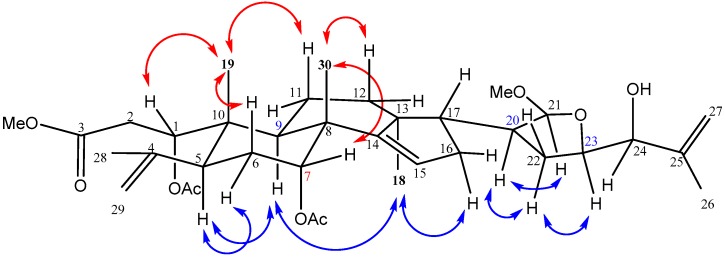
Key NOESY correlations of **1**.

The relative configuration of **1** was determined by the analysis of NOESY correlations (Figure 3). Assuming that H-5 of **1** was α-oriented similar to that of the A-seco apotirucallane tetranortriterpenes [15,29], the NOESY correlations between H-6α/H-5/H-9/H_3_-18/H-16α revealed that these protons were on the #x3B1;-face. The NOESY correlations of H_3_-19 with H-1, H-6β and H-11β, as well as Me-30 with H-7 indicating these protons were on the β-face. In addition, NOESY correlations of H-20/H-21/H-22α/H-23 suggested that these protons were all in α-face and the C-21 methoxy was β-oriented. On comparing the ^1^H- and ^1^^3^C-NMR spectra of **1** with those of chisopanin C [30] and polystanins C and D [31], it was noted that the configurations of C-20, C-21 and C-23 were assigned *S*, *S* and *R*, respectively, the same as those of polystanin D [31], and the hydroxy at C-24 was assigned as α-disposition, the same as that of chisopanin C [30]. Therefore, structure **1** was established and a name aphataiwanin A was given.

Compound **2**, ${\left[\alpha \right]}_{D}^{25}$ −90 (MeOH), had the same molecular formula C_36_H_54_O_9_ as **1**, as deduced from HRESIMS (*m/z* 653.3681 [M + Na]^+^) and DEPT spectra. The IR absorption bands revealed the presence of OH (3484 cm^−1^), ester (1740 cm^−1^) and double bond (1643 cm^−^^1^) in **2**. The 1D and 2D NMR spectra of **2** were similar to those of **1**, suggesting that **2** was an analogue of **1**. On comparing the ^1^^3^C-NMR spectra of **1** and **2**, it was found that the C-21 and C-17 chemical shifts of **2** (δc 109.3, 58.1) were downfield compared to the same carbons of **1** (δ 104.6, 52.9). Thus it was suggested that the configuration of the C-21 methoxy group in **2** was different from that in **1**. Also, both the COSY and HMBC correlations showed that all structural fragments were similar to those of **1**, confirming that compound **2** is an epimer of **1**, in which the C-21 methoxy was α-oriented.

The relative configuration of **2** was determined by NOESY experiment, in which the H_3_-19 and H_3_-30 were assigned to be β-oriented while H_3_-18, H-9, and H-5 were α-orientation. NOESY correlations between H-21/H-22β and H-20/H-23, and H-23/H-22β indicated that the methoxy group was α-oriented. Comparing the ^13^C-NMR data with those of chisopanin C [30] assigned the configuration of C-13, C-17, C-20, C-23 and C-24 as *S*, *S*, *S*, *R*, *R* and *R*, respectively. Therefore, the structure of compound **2** was determined and it was named aphataiwanin B.

Compound **3**, ${\left[\alpha \right]}_{D}^{25}$ −60 (MeOH), was obtained as an amorphous solid and found to possess the molecular formula C_36_H_56_O_10_, (one more oxygen atom than **1**) as inferred from its HRESIMS (*m/z* 671.3785 [M + Na]^+^). The similar ^1^H- and ^13^C-NMR spectroscopic data of **3** and **1** suggested that they are close analogues. However, the ^1^H-NMR spectrum of **3** exhibited eight methyl singlets (δ_H_ 0.95, 1.00, 1.14, 1.26, 1.27, 1.78, 1.96 and 2.04) instead of seven in **1** and only three olefinic protons (δ_H_ 4.84, 5.02 and 5.27) instead of five in **1**, implying that a double bond was missing in **3**. In the ^13^C-NMR spectrum, compound **3** was found possessing an additional methyl (δ_C_ 26.5) and lacking an olefinic methylene, in comparison to **1**. The additional methyl group was assigned at C-27 by observation of the HMBC correlations from H_3_-27 (δ_H_ 1.26) and H_3_-26 (δ_H_ 1.27) to C-25 (δ_C_ 73.1) and C-24 (δ_C_ 76.8). The relative configuration of **3** was determined on the basis of the NOESY experiment and comparing the *J* values of **3** with those of **1**. The result was identical to **1**, suggesting the same configuration. On the basis of above interpretations, the structure of **3** was categorized into the group of ring A-seco apotirucallol and a name aphataiwanin C was given.

Compound **4** was isolated as an amorphous solid, ${\left[\alpha \right]}_{D}^{25}$ −170 (MeOH). It had the same molecular formula C_36_H_56_O_10_ as **3**, as derived from HRESIMS at *m/z* 671.3763 ([M + Na]^+^). The IR spectrum revealed that **4** contained a hydroxyl (3491 cm^−^^1^), ester (1741 cm^−^^1^), and double bond (1642 cm^−^^1^) functionalities. The ^1^H- and ^13^C-NMR spectra of **4** (Table 1 and Table 2) were similar to those of **3** except for the hemiacetal carbon shifted downfield to δ_C_ 109.7 (C-21) in **3** and the oxygenated carbon C-23 shifted upfield to δ_C_ 76.9. The ^1^H-^1^H COSY and HMBC of **4** revealed cross peaks similar to those of **3**, suggesting that compound **4** was an epimer of **3**. The configuration at C-21 was determined by NOESY experiment. NOESY correlations between H-21/H-22β, H-20/H-22α and H-22α/H-23 indicated that the methoxy group was α-oriented. Other NOESY correlations were the same as those of **3**. Thus, the structure of compound **4** was elucidated and the name aphataiwanin D was given.

The 11 isolates were evaluated for their cytotoxic activities against human hepatocellular carcinoma (Hep-G2), lung carcinoma (A549), epithelial type 2 (HEp-2), and breast adenocarcinoma (MCF-7) cell lines in vitro. Among these compounds, **5** possessed significant activity (ED_50_ value of 6.83 ± 0.63 μg/mL) against MCF-7 cells and mild activity against Hep-G2 and A549 cells (ED_50_ value of 11.38 ± 0.98 and 15.49 ± 0.76 μg/mL). Simultaneously, compounds **1** and **10** also showed mild activities against Hep-G2 and HEp-2, respectively, with IC_50_ values of 16–17 μg/mL (Table 3). In terms of anti-inflammation, compounds **1**–**11** were tested on superoxide anion generation and elastase release by human neutrophils in the presence of FMLP/CB (Table 4). Only compounds **4** and **5** showed significant anti-inflammatory activity, as tested on superoxide anion generation with IC_50_ at 5.79 ± 0.88 and 1.25 ± 0.17 μg/mL, as well as the significant inhibition on elastase release with IC_50_ at 5.22 ± 0.24 and 2.26 ± 0.05 μg/mL, respectively.

## 6. Conclusions

In the present study, 10 tetranortriterpenoids, including four new compounds and a steroid, were isolated; compounds **1**–**5** belong to a group of protolimonoids, ring A-seco apotirucallol, while **6**–**10** can be classified as rings A,B-seco prieurianins. Among these secondary metabolites, liaphataiwanins A (**1**) and D (**4**), **5**, and prieurianin (**10**) were found to have mild cytotoxicities against cancer cells and moderate to potent anti-inflammatory activities. Compound **5**, which has the moiety of six-member ether ring, showed the best biological function. The chemical constituents and the evaluation of cytotoxicities against human cancer cells and anti-inflammatory activities reported herein may provide beneficial information for further phytotherapy research.

## Figures and Tables

**Table 1:** ^1^H-NMR spectroscopic data (400 MHz) of compounds **1**–**4** ^a^.

Position	1	2	3	4
1	5.49 d (10.8)	5.49 d (10.8)	5.49 d (10.8)	5.48 d (10.8)
2α	2.47 dd (13.8, 10.8)	2.46 dd (13.8, 10.8)	2.46 dd (13.8, 10.8)	2.46 dd (13.8, 10.8)
2β	2.81 d (13.8)	2.81 d (13.8)	2.81 d (13.8)	2.81 d (13.8)
5	2.44 d (3.0)	2.45 d (3.0)	2.44 overlap	2.44 d (3.6)
6*α*	1.63 dt (14.4, 3.0)	1.62 dt (14.4, 3.0)	1.63 d (13.8)	1.63 dd (13.8, 3.6)
6β	2.14 d (14.4)	2.14 d (14.4)	2.14 d (13.8)	2.15 d (13.8)
7	5.16 s	5.15 s	5.15 s	5.14 s
9	2.18 m	2.20 dd (12.0, 6.6)	2.19 dd (12.0, 6.6)	2.19 dd (12.0, 6.6)
11α	1.89 m	1.92 dd (12.0, 3.0)	1.94 d (12.0)	1.90 overlap
11β	1.67 m	1.66 m	1.70 m	1.67 dd (15.0, 12.0)
12α	1.65 m	1.86 dd (10.2, 3.0)	1.66 d (13.2)	1.81 dd (10.8, 9.0)
12β	1.54 dt (13.2, 9.6)	1.48 d (10.2)	1.55 dd (13.2, 9.6)	1.46 dd (10.8, 8.4)
15	5.27 d (3.0)	5.25 d (2.4)	5.27 d (2.4)	5.25 s
16α	1.93 dd (13.8, 7.8)	1.93 overlap	1.99 d (11.4)	1.92 d (15.6)
16β	2.10 ddd (13.8, 7.8, 3.0)	2.1 ddd (15.6, 7.2, 3.6)	2.10 m	2.08 dd (15.6, 7.8)
17	1.92 m	1.70 dd (7.2, 3.6)	1.97 m	1.71 dd (10.2, 7.8)
18	1.00 s	1.03 s	1.00 s	1.02 s
19	0.96 s	0.96 s	0.95 s	0.95 s
20	2.21 m	2.35 m	2.19 overlap	2.31 ddd (10.2, 9.6, 3.6)
21	4.78 d (4.2)	4.84 d (3.6)	4.76 d (4.8)	4.80 d (3.6)
22α	1.87 m	1.89 dd (12.6, 10.8)	1.89 overlap	1.87 m
22β	1.70 m	1.35 dd (12.6, 5.4)	1.86 dd (12.0, 7.8)	1.79 overlap
23	4.23 ddd (9.0, 6.6, 4.8)	4.08 ddd (10.8, 6.0, 5.4)	4.43 t (7.8)	4.25 dd (10.2, 4.2)
24	3.83 d (4.8)	3.92 d (6.0)	3.17 s	3.24 s
26	1.78 s	1.78 s	1.27 s	1.27s
27	5.05 s	5.00 s	1.26 s	1.26 s
27	4.93 s	4.91 s		
28	1.78 s	1.78 s	1.78 s	1.77 s
29	4.85 s	4.85 s	4.84 s	4.84 s
29	5.02 s	5.02 s	5.02 s	5.01 s
30	1.14 s	1.14 s	1.14 s	1.13 s
1-OAc	2.04 s	2.04 s	2.04 s	2.03 s
7-OAc	1.96 s	1.96 s	1.96 s	1.95 s
3-OCH_3_	3.66 s	3.66 s	3.65 s	3.65 s
21-OCH_3_	3.39 s	3.41 s	3.38 s	3.39 s

^a^ Chemical shifts are in ppm; *J* values in Hz are in parentheses; Measured in CDCl_3_.

**Table 2:** ^13^C-NMR spectroscopic data (100 MHz) of compounds **1**–**4** ^a^.

Position	1	2	3	4
1	77.2 CH	76.9 CH	76.8 CH	76.9 CH
2	35.5 CH_2_	35.5 CH_2_	35.5 CH_2_	34.9 CH_2_
3	172.0 C	172.1 C	172.0 C	172.1 C
4	145.1 C	145.0 C	145.1 C	145.0 C
5	44.3 CH	44.3 CH	44.3 CH	44.3 CH
6	29.3 CH_2_	29.3 CH_2_	29.3 CH_2_	29.3 CH_2_
7	74.7 CH	74.6 CH	74.7 CH	74.6 CH
8	42.5 C	42.5 C	42.4 C	42.4 C
9	34.5 CH	34.5 CH	34.4 CH	34.0 CH
10	44.3 C	44.3 C	44.3 C	44.3 C
11	18.5 CH_2_	18.4 CH_2_	18.5 CH_2_	18.4 CH_2_
12	33.5 CH_2_	33.7 CH_2_	33.5 CH_2_	33.4 CH_2_
13	46.3 C	46.6 C	46.2 C	46.6 C
14	159.2 C	159.5 C	159.1 C	159.4 C
15	119.1 CH	118.7 CH	119.2 CH	118.7 CH
16	35.2 CH_2_	35.0 CH_2_	35.2 CH_2_	34.4 CH_2_
17	52.9 CH	58.1 CH	52.7 CH	57.9 CH
18	20.8 CH_3_	20.2 CH_3_	20.8 CH_3_	20.2 CH_3_
19	15.2 CH_3_	15.2 CH_3_	15.2 CH_3_	15.2 CH_3_
20	45.8 CH	47.0 CH	45.0 CH	46.1 CH
21	104.6 CH	109.3 CH	104.9 CH	109.7 CH
22	31.0 CH_2_	34.2 CH_2_	31.6 CH_2_	33.6 CH_2_
23	80.7 CH	79.0 CH	79.0 CH	76.9 CH
24	78.1 CH	78.4 CH	76.8 CH	75.7 CH
25	144.9 C	144.6 C	73.1 C	73.3 C
26	18.7 CH_3_	18.4 CH_3_	26.6 CH_3_	26.6 CH_3_
27	112.8 CH_2_	113.6 CH_2_	26.5 CH_3_	26.5 CH_3_
28	23.0 CH_3_	23.0 CH_3_	23.0 CH_3_	23.0 CH_3_
29	116.6 CH_2_	116.6 CH_2_	116.6 CH_2_	116.6 CH_2_
30	26.9 CH_3_	26.9 CH_3_	26.9 CH_3_	26.9 CH_3_
1-OAc	170.5 C	170.5 C	170.5 C	170.5 C
	21.2 CH_3_	21.2 CH_3_	21.2 CH_3_	21.2 CH_3_
7-OAc	170.2 C	170.2 C	170.2 C	170.2 C
	21.5 CH_2_	21.5 CH_2_	21.5 CH_2_	21.4 CH_2_
3-OCH_3_	52.3 CH_3_	52.3 CH_3_	52.2 CH_3_	52.3 CH_3_
21-OCH_3_	54.9 CH_3_	55.7 CH_3_	55.4 CH_3_	55.9 CH_3_

^a^ Assignments were made using HMQC and HMBC techniques; Measured in CDCl_3_.

**Table 3:** Cytotoxic activities of compounds **1**–**11** ^a^.

Compound	ED_50_ (μg/mL)			
	HEp-2	Hep-G2	A549	MCF-7
**1**	28.12 ± 0.60	16.02 ± 0.41	33.56 ± 0.92	>40
**2**	36.05 ± 2.78	24.86 ± 1.70	>40	>40
**3**	37.78 ± 0.43	30.34 ± 0.51	>40	>40
**4**	37.72 ± 0.94	>40	>40	>40
**5**	11.38 ± 0.98	26.72 ± 1.01	15.49 ± 0.76	6.83 ± 0.63
**6**	>40	>40	>40	33.78 ± 1.66
**7**	>40	>40	>40	37.49 ± 1.98
**8**	31.06 ± 2.89	>40	>40	>40
**9**	36.69 ± 2.30	34.36 ± 3.43	> 40	>40
**10**	16.77 ± 2.36	>40	>40	>40
**11**	>40	>40	>40	>40
mitomycin	0.16± 0.01	0.19± 0.01	0.19± 0.01	0.18± 0.01

^a^ Results are presented as mean ± S.E.M. (*n* = 3).

**Table 4:** Anti-inflammatory data of compounds **1**–**11** ^a^.

Compound	Superoxide Anion		Elastase Release	
	IC_50_ (μg/mL) ^b^	Inh % ^c^	IC_50_ (μg/mL) ^b^	Inh % ^c^
**1**	>10	41.94 ± 2.39	>10	41.72 ± 4.47
**2**	>10	43.00 ± 4.46	>10	44.83 ± 5.06
**3**	>10	33.20 ± 6.23	>10	16.54 ± 3.36
**4**	5.79 ± 0.88	74.39 ± 2.90	5.22 ± 0.24	78.36 ± 5.74
**5**	1.25 ± 0.17	86.65 ± 1.75	2.26 ± 0.05	78.19 ± 0.56
**6**	>10	18.40 ± 0.84	>10	7.93 ± 3.27
**7**	>10	13.71 ± 3.60	>10	18.85 ± 2.47
**8**	>10	45.60 ± 3.63	>10	21.39 ± 3.77
**9**	>10	43.92 ± 7.10	>10	8.49 ± 1.05
**10**	>10	49.20 ± 2.20	>10	39.36 ± 4.48
**11**	>10	44.85 ± 1.63	>10	33.64 ± 8.34

^a^ Results are presented as mean ± S.E.M. (*n* = 3); ^b^ Concentration necessary for 50% inhibition (IC_50_); ^c^ Percentage of inhibition (Inh %) at 10 μg/mL concentration.
